# CML derived exosomes promote tumor favorable functional performance in T cells

**DOI:** 10.1186/s12885-021-08734-3

**Published:** 2021-09-07

**Authors:** Nazli Jafarzadeh, Mohammad Ali Gholampour, Mohammad-Reza Alivand, Saeideh Kavousi, Laleh Arzi, Fariba Rad, Majid Sadeghizadeh, Majid Pornour

**Affiliations:** 1 Department of Genetics, Faculty of Biological Sciences, Tarbiat Modares University, Tehran, Iran; 2 Department of Hematology, Faculty of Medical Science, Tarbiat Modares University, Tehran, Iran; 3 Department of Medical Genetics, Faculty of Medicine, Tabriz University of Medical sciences, Tabriz, Iran; 4 Department of Medical Genetics, Faculty of Medical Sciences, Tarbiat Modares University, Tehran, Iran; 5 Department of Microbiology, Shahr-e-Qods Branch, Islamic Azad University, Tehran, Iran; 6 Cellular and Molecular Research Center, Yasuj University of Medical Sciences, Yasuj, Iran; 7 Department of Photo Healing and Regeneration, Medical Laser Research Center, Yara Institute, ACECR, Tehran, Iran

**Keywords:** Human primary cord blood T cell, Chronic myelogenous leukemia (CML)-derived, Exosomes, Immunosuppression, Tumor microenvironment

## Abstract

**Background:**

Leukemic cells facilitate the creation of the tumor-favorable microenvironment in the bone marrow niche using their secreted factors. There are not comprehensive details about immunosuppressive properties of chronic myelogenous leukemia-derived exosomes in the bone marrow stromal and immune compartment. We explained here that K562-derived exosomes could affect the gene expression, cytokine secretion, nitric oxide (NO) production, and redox potential of human primary cord blood-derived T cells (CB T cells).

**Methods:**

Human primary cord blood-derived T cells were treated with K562-derived exosomes. We evaluated the expression variation of some critical genes activated in suppressor T cells. The alterations of some inflammatory and anti-inflammatory cytokines levels were assessed using ELISA assay and real-time PCR. Finally, NO production and intracellular ROS level in CB T cells were evaluated using Greiss assay and flow cytometry, respectively.

**Results:**

Our results showed the over-expression of the genes involved in inhibitory T cells, including NQO1, PD1, and FoxP3. In contrast, genes involved in T cell activation such as CD3d and NFATc3 have been reduced significantly. Also, the expression of interleukin 10 (IL-10) and interleukin 6 (IL-6) mRNAs were significantly up-regulated in these cells upon exosome treatment. In addition, secretion of the interleukin 10, interleukin 6, and interleukin 17 (IL-17) proteins increased in T cells exposed to K562-derived exosomes. Finally, K562-derived exosomes induce significant changes in the NO production and intracellular ROS levels in CB T cells.

**Conclusions:**

These results demonstrate that K562-derived exosomes stimulate the immunosuppressive properties in CB-derived T cells by inducing anti-inflammatory cytokines such as IL-10, reducting ROS levels, and arising of NO synthesis in these cells. Moreover, considering the elevation of FOXP3, IL-6, and IL-17 levels in these cells, exosomes secreted by CML cells may induce the fates of T cells toward tumor favorable T cells instead of conventional activated T cells.

**Supplementary Information:**

The online version contains supplementary material available at 10.1186/s12885-021-08734-3.

## Introduction

Chronic myelogenous leukemia (CML) is one of the hematological disorders in adults with an incidence of 1–2 cases per 100,000. Approximately 15% of all newly diagnosed leukemia cases in adults are CML. Estimations of CML prevalence in the united states indicates 30,000 in 2000 and increased to near about 150–180,000 in 2020 [1]. The development of CML occurs due to the continuous expression of a fusion onco-kinase BCR-ABL due to chromosomal translocation t(9:22), which leads to the uncontrolled proliferation and inhibition of apoptosis in hematopoietic progenitor cells within the bone marrow [1, 2].

Current evidence indicates that leukemia's progression is dramatically related to dynamic crosstalk between leukemia cells and the host immune system [3, 4]. Malignant cells have the ability to escape immune surveillance by some strategies in which cell-secreted factors are among the most important strategies applied by tumors to attenuate host immune attacks [3].

Exosomes are nano-sized membrane-bound vesicles (approximately 30–200 nm in diameter) and originate from the endocytic pathway. These types of extracellular vesicles are secreted by almost all cell types (normal and malignant cells) from various histological origins and play a prominent role in cellular trafficking and communications [5–7].

All exosomes share many characteristics in common, including the size, specific marker expression profile (CD9, CD63, CD81, etc.), and the ability to transfer molecular messages to their surrounding microenvironment or distant tissues. Furthermore, exosomes can represent the specific molecular characteristics of their original mother cells [5, 7, 8]. During the past few years, considerable attention is being directed to the tumor-derived exosomes and their role in tumor progression, evasion, and immune suppression [9].

Particularly, considerable studies have been conducted to address the role of exosomes derived from different types of leukemia on tumor progression, angiogenesis, and niche reprogramming, previously [3]. Our previous study supported this notion. We explored the possible role of K562 CML cell line-derived exosomes on the main cell sources in bone marrow stroma (mesenchymal stem cells and macrophages). Given this report, we found that CML cell-derived exosomes could induce a microenvironmental shift in BM niche cells toward a more immunosuppressive and tumor progressive niche [10].

During the last years, there is much interest in identifying the role of tumor-derived exosomes on T cell function as the main cellular component in the immune system [11–14]. To the extent of our knowledge, there is limited knowledge regarding the exact role of CML-derived exosomes on the functional alterations of T lymphocytes. Hence, this study has been designed to explore the regulatory roles of CML-derived exosomes on the critical immunological properties in primary human cord blood (CB)-derived T lymphocytes. Our results may provide new evidence into the possible role of CML-derived exosomes in the host immune system modulation.

## Materials and methods

### Cell culture and reagents

The human chronic myelogenous leukemia cell line (K562) was purchased from the Pasteur Institute of Iran and maintained in a growth medium containing RPMI 1640 supplemented with 10% fetal bovine serum (FBS) (Gibco, Australia), 100 mg/mL streptomycin (Sigma-Aldrich, USA), and 100 U/mL penicillin (Sigma-Aldrich, USA) (at 37 °C and humidified 5% CO2 atmosphere).

Human primary CB T cells were isolated by the nylon wool method. The Preservative CB samples that contained citrate phosphate dextrose (CPD) anti-coagulant were collected from the Iranian Blood Transfusion Organization (IBTO) Public Umbilical Cord Blood Center, Tehran, Iran during 2017–2018. Red cells were initially precipitated using 6% hydroxyl-ethyl- starch (HES, Spain). The suspended cells were centrifuged and layered over Ficoll-Hypaque 1.077 (Lymphodex, Germany). The MNC layer was collected from the supernatant by density gradient centrifugation. Next, MNCs were cultured in DMEM (Gibco, USA) supplemented with 10% FBS. After 3 h, adherent cells such as monocytes are depleted by adhering over flask cell culture surface, and suspended cells are enriched for lymphocytes. The suspended cells were incubated and passed through a nylon wool column as previously described [15]. B lymphocytes were trapped into the column, although T cells were passed after aspiration by RPMI medium. The collected cells were analyzed by flow cytometry panel for surface marker expression including CD3, CD45RA, and CD197 using mouse anti-human CD3 (Abcam, USA), mouse anti-human CD45RA (Abcam, USA), and mouse anti-human CD197 (BD Biosciences, USA) antibodies. Isotype control antibodies were used as a negative control for excluding nonspecific binding. The data analyses were carried out using the FlowJo software v.7.6.1.

### Exosome isolation

The K562 cells were grown in Exosome-depleted media (RPMI 1640 supplemented with 10% exosome-free FBS), and conditioned media was collected every 48 h. The extracellular vesicles (EVs) were depleted from FBS by an ultracentrifugation-based protocol. The FBS was centrifuged twice for 2 h at 120000 g, and the supernatant was passed through 0.22 μm PVDF or PTFE filter meshes before cell culture. The exosome isolation was performed using ultracentrifugation based on the protocol that we used in our previous study [10, 16]. First, conditioned media differentially centrifuged for 30 min at 300 g, 30 min at 2000 g, and 60 min at 10000 g to  remove live cells, dead cells, and small debris. Then, the supernatant obtained from centrifugation was subjected to an extra centrifugation step for 120 min at 20000 g to remove larger vesicles such as microvesicles. Finally, exosomes were isolated by 120 min centrifugation at 100000 g. The exosome pellet was suspended in phosphate-buffered saline (PBS) and stored at − 80 °C for downstream analysis. The protein content of isolated exosomes was determined using Bradford assay according to manufactures instructions.

### Immunoblotting

To confirm the presence of exosomes, we determined the level of exosomes surface markers (CD81, CD63, and CD9) in K562-derived exosomes compared to K562 whole cell lysate. Briefly, 20 μg of each exosome preparation and K562 cell lysate (extracted using RIPA buffer supplemented with a cocktail of protein inhibitors) were loaded onto 14% acrylamide SDS–PAGE gels electrophoresis. The blots were probed with a primary mouse anti-human CD81 antibody (Santa Cruz, USA), a primary mouse anti-human CD9 antibody (Santa Cruz, USA), and a primary mouse anti-human CD63 antibody (Santa Cruz, USA). The membranes were washed 3x with TBST and incubated with an HRP-conjugated anti-mouse secondary antibody for 1 h. Finally, the protein expression level was detected using an ECL Western blotting substrate (ECL, Amersham, Buckinghamshire, UK).

### Transmission electron microscopy (TEM)

A total of 20 μg of the fresh exosome was pipetted onto a carbon Film 300 mesh cooper grid (AGS160–3) and incubated for 5 min. The resultant grid was rinsed with 20 μL of sterile distilled water. Then, the exosome coated grid was negatively stained with 1% uranyl acetate for 2 min. Finally, the grids were placed in a clean petri dish and transferred for transmission electron microscopy (TEM) imaging. The prepared samples were analyzed with a Zeiss-EM10C transmission electron microscope at a voltage of 80 kV.

### Scanning electron microscopy (SEM)

The morphology of the exosomes was observed using scanning electron microscopy (SEM) to evaluate the nanostructure of vesicles. A total of 20 μL of the fresh K562-derived exosomes sample was pipetted onto a glass slide and incubated for 2 min. Then, the slides were placed in a clean petri dish and transferred for SEM imaging. The resultant slides were gold-coated before imaging. The images were acquired using a KYKY-EM3200 scanning electron microscope at 26 kV.

### Size measurement of exosomes by dynamic light scatter

The size distribution of purified exosomes was measured using Dynamic Light Scattering (DLS). Briefly, the exosome samples were loaded into a quartz cuvette and measured by a Malvern Nano ZS instrument (Malvern instrument, UK) at 25 °C. Finally, the results were analyzed using Zetasizer software (version 7.11).

### Exosomes uptake assay

According to the manufacturer's protocol, the fluorescent labeling of the purified exosomes was performed using a PKH26 Fluorescent Cell Linker Kit (Sigma-Aldrich, St Louis, MO). K562 cell-derived exosomes were fluorescently labeled with PKH26 membrane labeling dye. 2 × 10^5^ of human cord blood T cells were seeded in 12-well tissue culture plates (in a humidified incubator, 37 °C). Then the fluorescently labeled exosomes were added to the culture media for 2 and 4 h. After that, the cells were fixed with 4% paraformaldehyde (Mojallali, Tehran, Iran), and the nuclei of T cells were stained with 4′,6-diamidino-2-phenylindole (DAPI; Sigma-Aldrich, St Louis, MO). Then, the cells were transferred on a coverslip, and the uptake of exosomes was imaged using a spectral Leica TCS SPE confocal microscope (Germany).

### RNA analysis and qRT-PCR

2 × 10^5^ of the cord blood T cells were seeded in 12-well culture plates and treated with 100 μg/ml of K562-derived exosomes for 72 h. Total RNA was extracted from 2 × 10^5^ cells using **TRIzol**™ Reagent (Ambion, USA). The concentration and purity of the RNA samples were assessed as 260/280 nm and 260/230 nm ratios, respectively. One microgram of total RNA was used for cDNA synthesis. cDNA was synthesized with a first-strand cDNA synthesis kit (Thermo Fisher Scientific, Waltham, MA). Quantitative RT-PCR was performed using SYBR Green master mix kit (Genaxxon kit, Germany) and Step One Real-time PCR system (Applied Biosystems, USA).

Relative changes in expression of selected genes between control and exosome treated groups were analyzed using the ΔΔCT method. Primer sets used for qRT-PCR are listed in Table 1.

**Table 1:** The primer sequences used in the current study

	Forward	Reverse
Gapdh	5′-CCGAGCCACATCGCACAG-3′	5′-GGCAACAATATCCACTTTACCAG-3′
Β-actin	5′-AGACGCAGGATGGCATGGG-3’	5′-GAGACCTTCAACACCCCAGCC-3’
TNF-α	5′-ATGTTGTAGCAAACCCTCAAGC-3′	5′-AGGACCTGGGAGTAGATGAGG-3′
TGF-β	5′- AGTGGACATCAACGGGTTCAC-3′	5′-TGGAGCTGAAGCAATAGTTGGTG-3′
IL-6	5′- GCACTGGCAGAAAACAACCT-3′	5′- CAGGGGTGGTTATTGCATCT-3′
IL-10	5′-TCTCCGAGATGCCTTCAGCAGA-3’	5′-TCAGACAAGGCTTGGCAACCCA-3’
IL-17	5′- CGGACTGTGATGGTCAACCTGA-3’	5′- GCACTTTGCCTCCCAGATCACA-3’
CD3d	5′-AAGTGAGCCCCTTCAAGATACC-3’	5′-TCTGAGAGCAGTGTTCCCAC-3’
ITK	5′-ACTCCTGAAGACAACAGGCGA-3′ ′	5′ATCCTTCATGCCCATTCCTGTC-3′
NFATC3	5′-GTGAAGCTCCTGGGCTATAACG-3′	5′-TATCTCTTGGCTTGCAGTAGCG-3′
NFATC4	5′-GAAGGGTGAGACGGACATCG-3′	5′-TTGGAGCCAGTCAGTACCAGT-3′
FOXO1	5′- GAGGGTTAGTGAGCAGGTTACAC-3’	5′- TGCTGCCAAGTCTGACGAAAG-3’
FOXP3	5′-TTCATCTGTGGCATCATCCGAC-3	5′- GTCGCATGTTGTGGAACTTGAAG-3′
PD-1	5′-CGTGGCCTATCCACTCCTCA-3’	5′-ATCCCTTGTCCCAGCCACTC-3’
NQO1	5′-CCTGCCATTCTGAAAGGCTGGT-3’	5′-GTGGTGATGGAAAGCACTGCCT-3’
C-myc	5′-CCTGGTGCTCCATGAGGAGAC-3’	5′-CAGACTCTGACCTTTTGCCAGG-3’

### Greiss assay

The amount of NO produced by cord blood T cells was estimated from the existence of nitrite (NO2-) as a stable metabolite of nitric oxide using Griess reaction assay. One hundred microliters of Griess reagent containing 1% of sulphanilamide and 0.1% of naphthylenediamine in 5% phosphoric acid (Merck, Germany) were mixed with an equal volume of culture supernatant from cell culture samples. The absorbance of each sample was measured at 540 nm on a plate reader immediately. The amount of NO production was expressed as micromole per milliliter. The NO levels were evaluated in supernatant of cord blood T cells at two concentrations of the exosome, including 50 μg/ml and 100 μg/ml after 72 h.

### Determination of intracellular reactive oxygen species (ROS)

Cellular ROS production was determined using 2,7-dichlorodihydrofluorescein diacetate (H2DCFDA, Sigma, USA). Cells were washed with PBS and incubated with 500 μl of 10 μM H2DCFDA (Sigma) solution (diluted in serum-free DMEM) in a dark and humidified 37 °C incubator for 45 min. After that, the cells were washed and analyzed with a FACS Calibur (Becton Dickinson, Bedford, MA). DCFDA is excited by the 488 nm laser and detected at 535 nm. The results of ROS flow cytometry were analyzed using FlowJo software (V.10).

### ElISA assay

Supernatants of the cells were centrifuged at 1500×g for 10 min at 4 °C to remove debris. Then, we determined the concentrations of IL-6, IL-10, IL-1β, IL-8, and TNF-α cytokine secretion levels by ELISA kits (International Brands Limited [IBL]), according to the manufacture’s protocols. Absorbance at 450 nm was measured using an ELISA plate reader (ELX800TM, USA).

### Statistical analysis

All statistical analysis was performed by GraphPad Prism (Version 6) and Excel 2013 (Microsoft). Significant differences between the two experimental groups were examined by Student’s t- test. Data are presented as mean ± SEM and *P*-values < 0.05 were considered to be statistically significant.

## Results

### Primary human cord blood T cells characterization

**Fig. 1:**
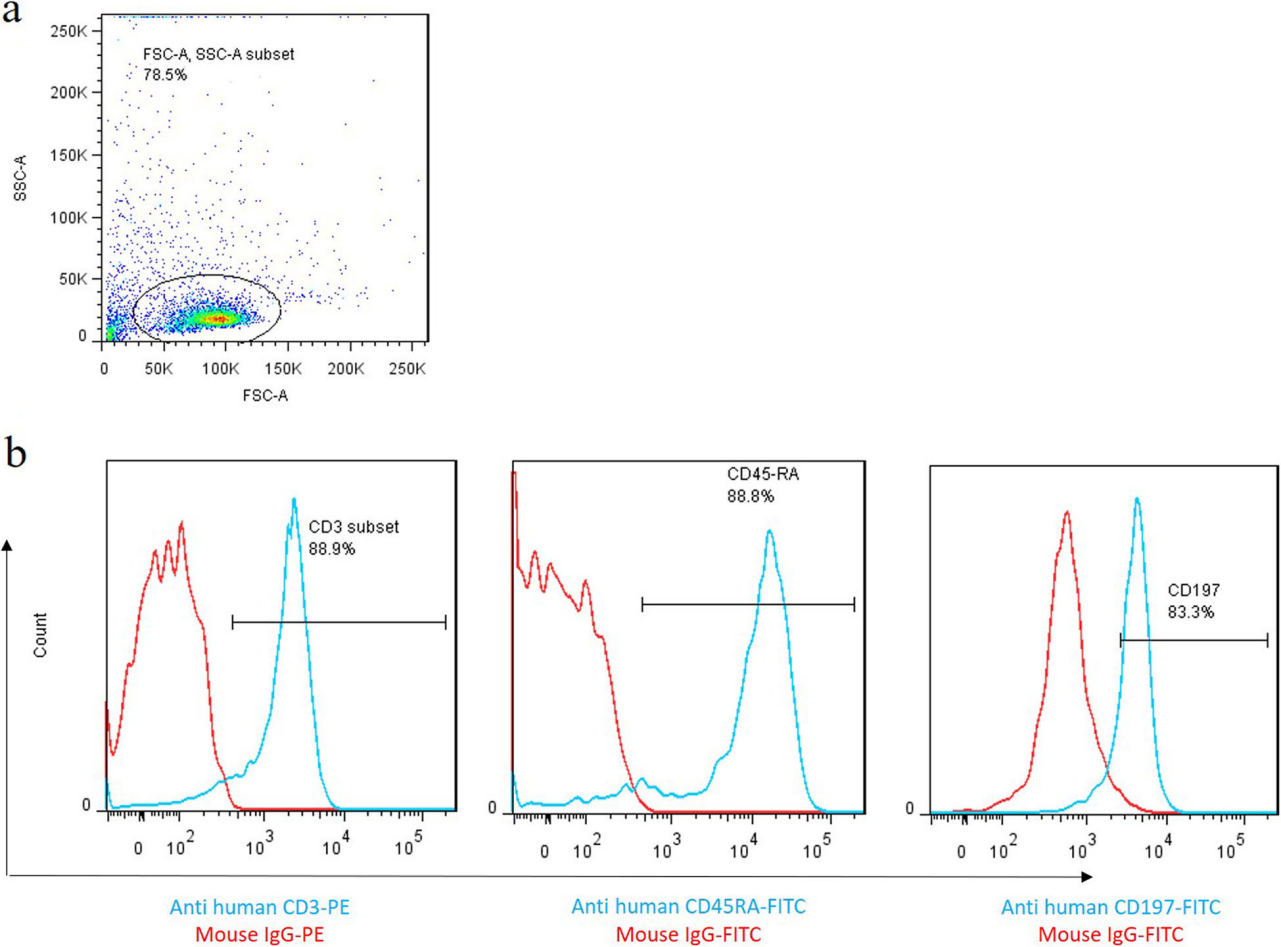
Characterization of cord blood naive T cells. Immunophenotypes of the T-cells were examined by flow cytometry. **a** Graph represented the cell population by using FSC-A versus SSC-A plot. **b** Overlaid histograms of CD3, CD45RA as well as CD197 expression. T cells were stained using surface staining protocol with specific fluorescent-conjugated antibodies, and the positive population was confirmedusing relevant isotype controls. Red histograms represent the isotype controls, and the blue histograms indicate CD3, CD45RA, and CD197-specific antibodies

The flow cytometry results indicated that the isolated cells were uniformly positive for T cell-specific markers (CD3+). These results confirmed that the homogenous population of T cells was isolated in the present study. Furthermore, flow cytometry results revealed that isolated CB T cells were positive for naïve T cells markers, including CD45RA and CD197 (Fig. 1).

### Characterization of K562-derived exosomes

**Fig. 2:**
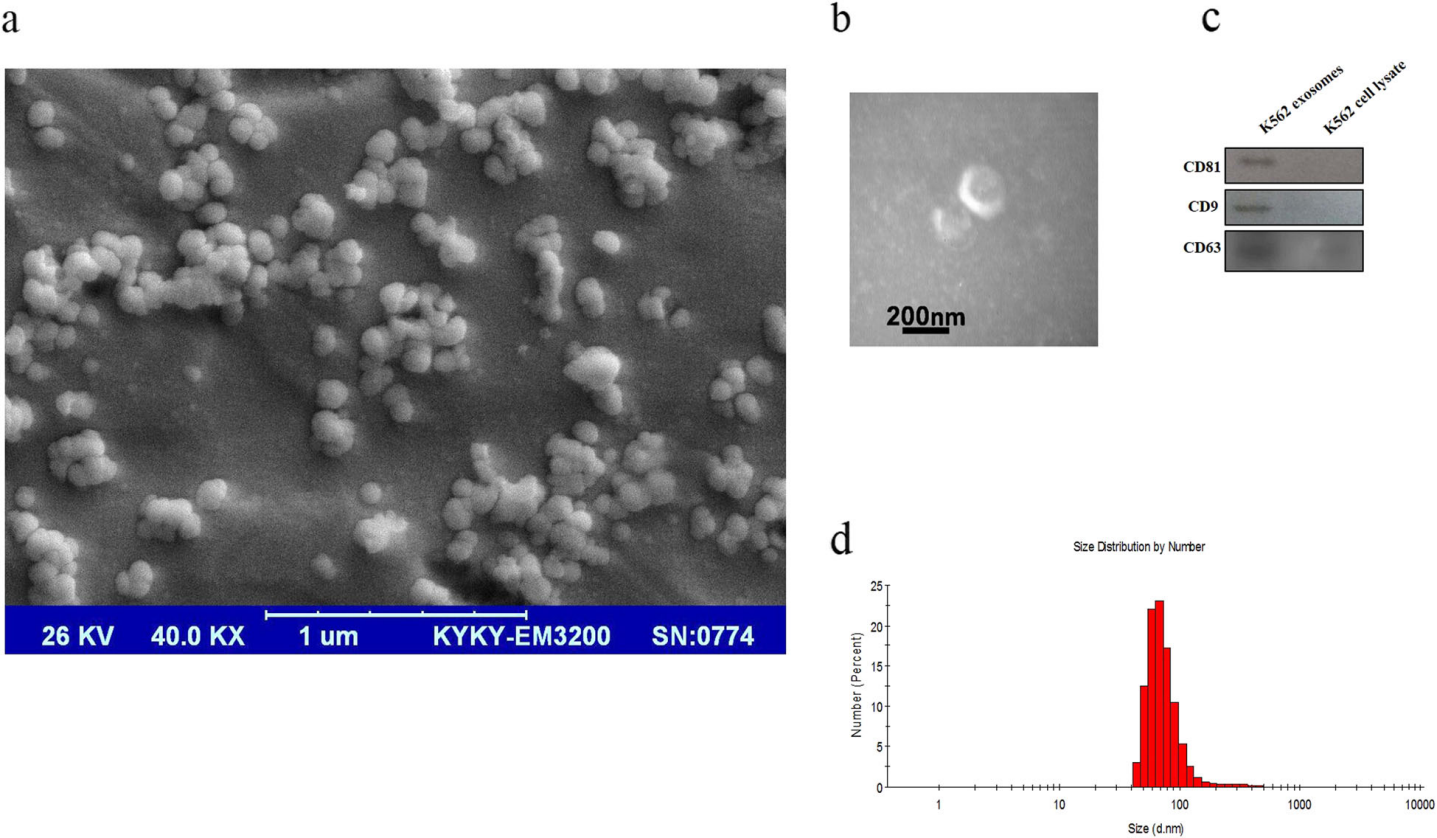
K562 CML cells secrete vesicles with the characteristics of exosomes in their culture media, **a** Scanning electron microscopy (SEM) image of purified exosomes. Vesicles were isolated from the cell culture supernatant of K562 cells and examined by scanning electron microscopy. **b** Transmission electron microscopy (TEM) of K562-derived exosomes, **c** Western blot analysis of the purified exosomes obtained using ultracentrifugation for exosome surface markers including CD81, CD9, and CD63. K562 cell lysate was used as a control. **d** Size analysis of K562-derived exosomes using dynamic light scattering (DLS)

The electron microscopy analysis (SEM and TEM) revealed that the harvested ultracentrifuged pellet contains membranous nano-sized particles with spherical morphology (Fig. 2a and b). The purified particles' size was also in the expected range for exosomes (50–200 nm; Fig. 2a). In addition, Western blot analysis confirmed that the exosomes were positive for the exosome-specific markers, including CD81, CD63 as well as CD9 (Fig. 2c). Complete and original figures of SEM and TEM derived exosomes besides original membranes showed exosomes markers presented as Figs. 1 and 2 in the supplementary file. The average size of exosomes was evaluated by DLS. The size distribution of isolated particles was in the range of 40–120 nm with the polydispersity index [PDI] of 0.353 (Fig. 2d).

### Cellular uptake of K562-derived exosomes by human cord blood T cells

**Fig. 3:**
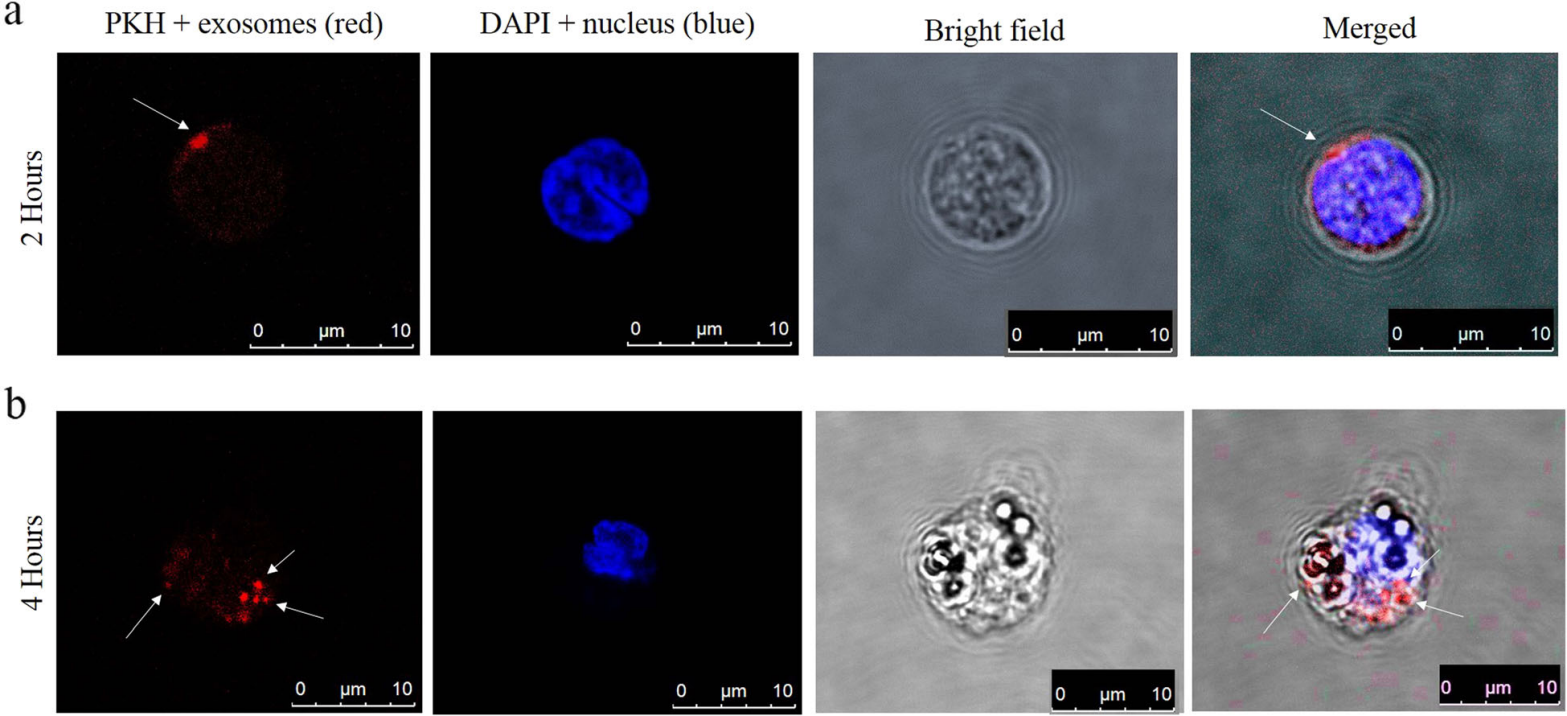
Confocal microscopy confirmed uptake and internalization of K562-derived exosomes (labeled with the PKH26 lipophilic dye) by recipient cells with nucleus staining by DAPI. **a** Uptake of K562-derived exosomes by cord blood T cells after 2 h incubation. **b** Uptake of K562-derived exosomes by cord blood T cells after 4 h incubation

We studied the internalization of K562-derived exosomes by human cord blood T cells; K562-derived exosomes were stained with the PKH dye and incubated for either 2 or 4 h with cord blood T lymphocytes in a humidified CO2 incubator (37 ^ͦ^ C). Then, the cells were fixed, and the nuclei of cells were stained with DAPI. Figure 3 shows the internalization and localization of red-labeled exosomes in the cytoplasm of the T cells. The confocal microscopy results indicated that fluorescently labeled exosomes could successfully incorporate and uptake by cord blood T cells (Fig. 3 a and b).

### K562-derived exosomes induce transcriptional and morphologic changes in cord blood T cells

**Fig. 4:**
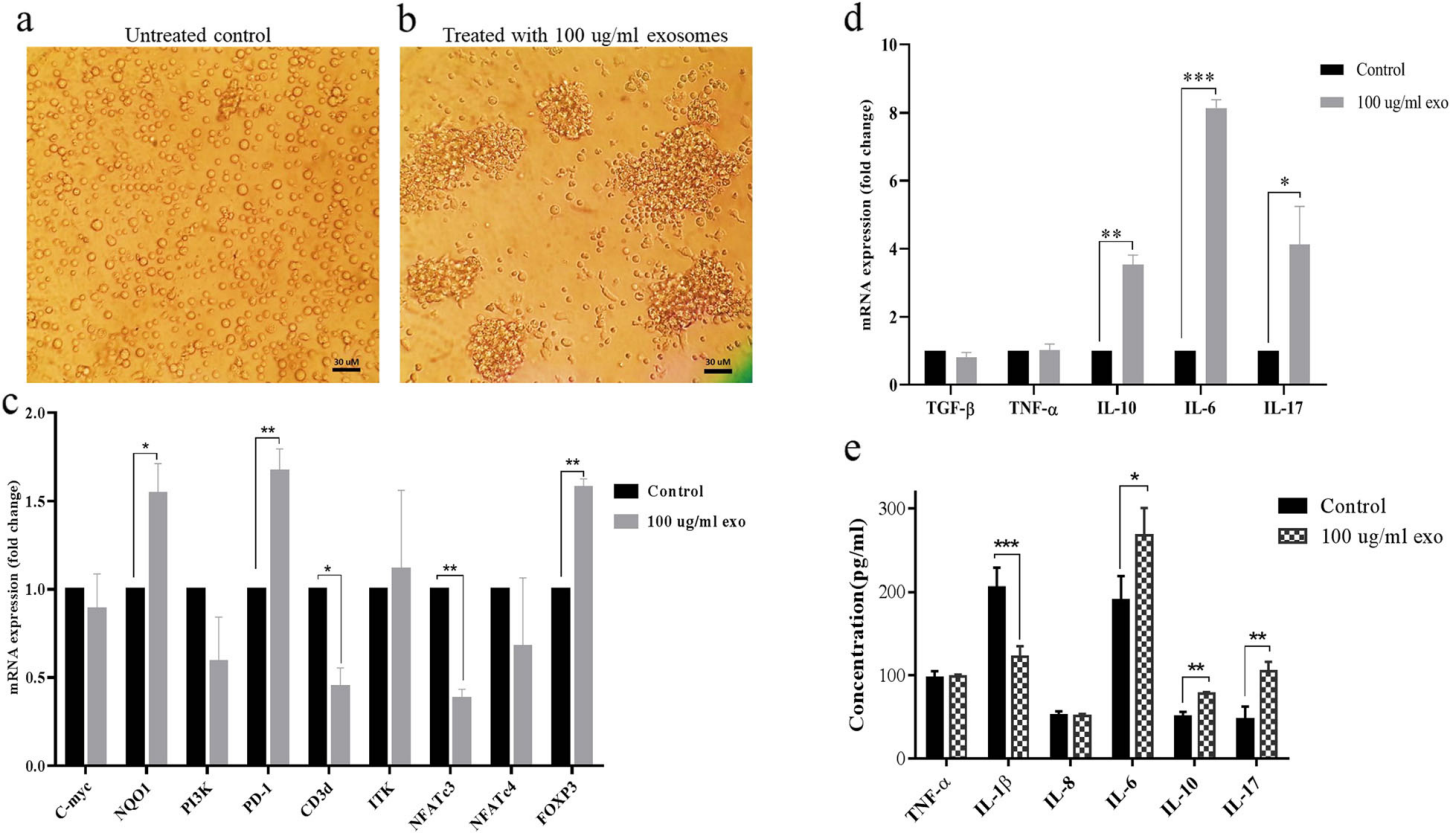
The effects of K562 derived exosomes on morphology, gene expression profile, and cytokine secretion of cord blood T cells, **a** Shows untreated control, **b** Shows the aggregation of cord blood T cells after treatment with 100 μg/ml concentration of K562 derived exosomes, **c** the effects of K562 derived exosomes on c-myc, NQO1, PI3K, PD1, CD3d, ITK, NFATc3, NFARc4, and FOXP3 expression, **d** The effects of K562 derived exosomes on the expression of TGF-β, TNF-α, IL-10, IL-6 and IL-17 in cord blood T cells, **e** The effects of K562 derived exosomes on TNF-α, IL-1β, IL-8, IL-6, IL-10 and IL-17 secretion from cord blood T cells. Data represented as mean ± SD (error bars) of three independent experiments in triplicate assays (***; *P*-value < 0.001, **; *P*-value < 0.01 and *; *P*-value < 0.05)

The morphology of exosome-treated CB T cells changed, and they were aggregated (Fig. 4a and b), which shows the promotion of T cell functional performance with the minimal phenotype differentiation [17].

In addition, K562 derived exosomes altered the expression level of genes involved in CB T cells polarization toward immune-suppressive phenotypes. Our qRT-PCR results demonstrated that the expression levels of NQO1 (1.54 folds, *P*-value < 0.05), PD1(1.7 folds, *P*-value < 0.01), and FoxP3 (1.6 folds, *P*-value < 0.01) increased significantly in treated CB T cells. Also, the expression of the c-myc gene was not changed. In contrast, treatment with 100 μg/ml of purified exosomes significantly decreased the expression level of CD3d (2.3 folds, *P*-value < 0.05) and NFATc3 (2.7 folds, *P*-value < 0.05) within 72 h (Fig. 4c).

However, such remarkable variation has not occurred for PI3K, ITK, and NFATc4 genes (Fig. 4c).

When the cord blood T cells were exposed to 100 μg/ml of exosomes for 72 h, the expression level of IL-10, IL-6, and IL-17 mRNAs were significantly up-regulated around 3.54 (*P*-value< 0.01), 8.13 (*P*-value< 0.001), and 4.2 ((*P*-value< 0.05) folds compared to untreated control. In this case, TNF-α and TGF-β did not have any significant changes (Fig. 4d).

### K562-derived exosomes induce significant changes in cytokine secretion level in cord blood T cells

The levels of inflammatory and anti-inflammatory cytokines, including TNF-α, IL-1β, IL-8, IL-10, IL-6, and IL-17, were determined in the supernatant of CB T cells after treatment by 100 μg/ml exosomes (Fig. 4e). The result of the ELISA assay showed that CML-derived exosomes significantly stimulated the expression level of IL-10, IL-6, and IL-17  proteins that were secreted in the conditioned medium of T cells treated with 100 μg/ml exosomes within 72 h. Also, a significant reduction was observed in the IL-1β level (Fig. 4e). However, no significant changes were observed in TNF-α and interleukin 8 levels (Fig. 4e).

### ROS production was moderated with K562-derived exosomes in cord blood T cells

**Fig. 5:**
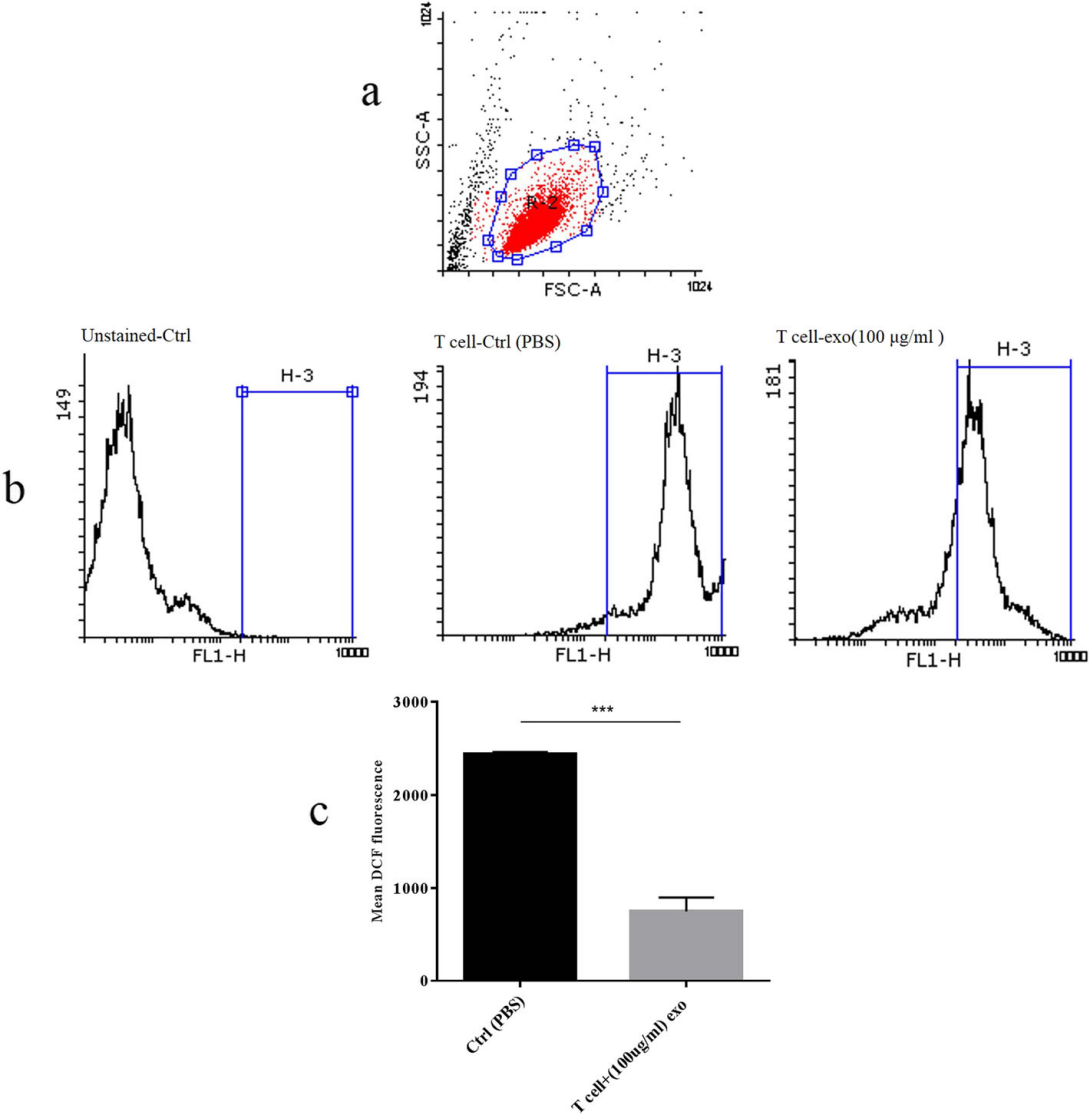
Flow cytometry analysis of intracellular ROS generation in cord blood T cells after 72 h treatment with K562-derived exosomes. **a** Graph represented the cell population by using FSC-A versus SSC-A plot. **b** Histograms show the comparison of intracellular DCF fluorescent intensity in the unstained control group, and control (PBS treated) group, and exosome-treated group, **c** Represented the comparison of ROS levels between the control (PBS treated) group and exosome-treated group. (***Significant at *p*-value < 0.001). ROS: reactive oxygen species; DCF: dichlorodihydrofluorescein; FSC-A: forward scatter-A; SSC-A: side scatter-A

Activation of T cells through ROS production has been shown in T cell studies, whereas IL-17 producing T cells induction has been observed in ROS reduction condition [18]. Interestingly, they explained that the intracellular redox balance could control the polarization of cord blood T cells in physiological and pathological conditions [18]. To elucidate the role of leukemia-derived exosomes in manipulating the intracellular ROS level in cord blood T cells, we measured the fluorescent signal of H2DCFA by flow cytometry. Our results showed that the exosomes treatment of CB T cells resulted in a significant reduction of the intracellular ROS level during 72 h culture period (Fig. 5a-c).

### K562-derived exosomes induce significant changes in the NO production level in cord blood T cells

**Fig. 6:**
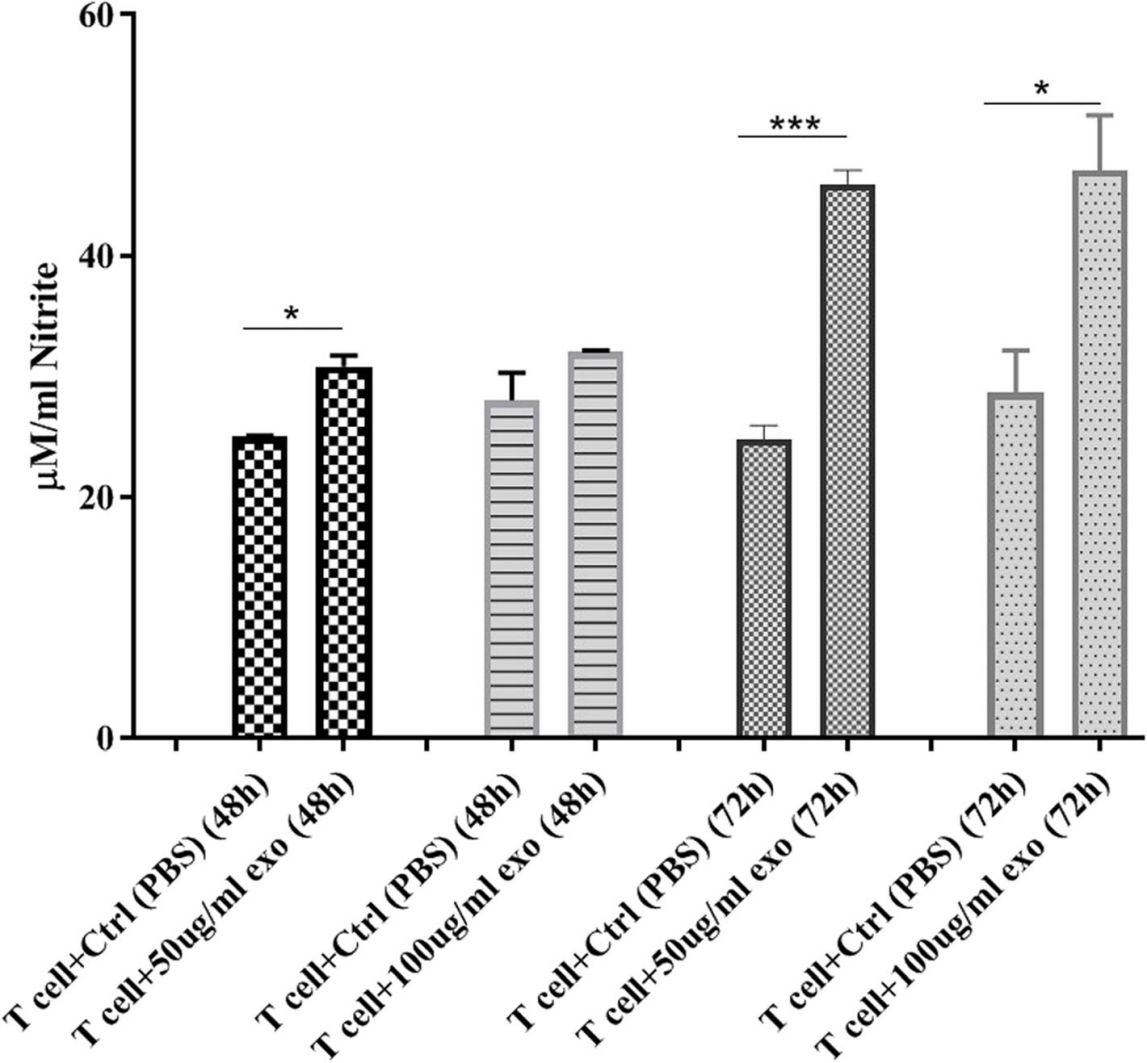
Effect of K562-derived exosomes on nitric oxide production in the supernatant of cord blood T cells after treatment with 50 μg/ml and 100 μg/ml at 48 and 72 h. (*Significant at *p*-value < 0.05, ***Significant at *p*-value < 0.001)

NO is an important mediator of cellular function, and its role in immunomodulation, angiogenesis, and tumor cell behavior is well documented [10, 19]. To examine the effect of K562-derived exosomes on the NO production in cord blood T cells, we measured the NO production after a 72 h treatment with 50 μg/ml and 100 μg/ml of K562-derived exosomes (Fig. 6). Assessment of NO production in CB T cells demonstrated that both 50 μg/ml and 100 μg/ml of exosomes induce a significant increase in NO secretion within 48 (50 μg/ml concentration of exosomes) and 72 h (both 50 μg/ml and 100 μg/ml concentration of exosomes), and the maximum elevation occurred in 50 μg/ml concentration of K562-derived exosomes at 72 h compared with the control group (Fig. 6).

## Discussion

We hypothesized that Chronic myelogenous leukemia can alter the molecular and functional properties of T cells. All of the evidence in the current study described how K562 derived exosomes may exert immunosuppressive effects in T cells, and how their secretions change the properties of these cells. The secretome of Chronic leukemic cells dictates the bone marrow microenvironment to generate a tumor favorable condition through making fundamental modifications in properties of key components of bone marrow niche including macrophages and BM-MSC [10]. As per our knowledge, BM-MSCs were changed in patients with CML and provided a tumor favorable microenvironment. A previous study indicated that macrophages were polarized to the tumor-associated M2-like macrophages in leukemia situations [10].

Different responses were observed in T cells exposed to tumor-derived exosomes (TDEs) from various cancer types, including head and neck cancer cells, melanoma cells, and prostate cancer cells [11]. For example, suppression of cytotoxic T cells, apoptosis of activated T cells, obtaining suppressor T cell behavior, and differentiation to regulatory T cells were the major variation of T cells affected by exosomes from different tumor types [11, 20, 21]. In our study, we observed that CB-derived T cells showed the properties of regulatory T cells after exposure to K562-derived exosomes. A significant increase in the level of FOXP3 and the elevation of IL-10 represented the properties alteration of these cells toward inhibitory or regulatory T cells.

Higher expression of PD-1 in T cells, as well as reduction of TCR/CD3 complex chains such as cd3d (delta chain) and ITK (as an adaptor protein), attenuate T cell receptor triggering signals [22–24]. Interaction between PD-1 and tumor cells PD-L1 can negatively influence T cell receptor triggering signals via recruitment of SHP2 to counteract with TCR/CD3 complex activation signals [22, 25]. PD-1 signaling is well-known for induceing clonal exhaustion or anergy of antigen-specific T cells during tumor formation and progression [26]. Elevation of PD-1 in CD8+ cells was reported in cancer patients, and the blockage of PD-1/PD-L1 interaction may help to restore the function of CML-specific cytotoxic T-lymphocytes (CTLs) [27]. In the current study, we observed that K562-derived exosomes decreased the CD3d expression and increased the expression of PD-1 in cord blood-derived T cells. Therefore, we could presume that CML-derived exosomes have the potency to weaken the antigen-mediated signal transduction pathway in T cells.

NFATc3 and NFATc4 are two members of the NFAT1–4 family which play crucial roles in activation, differentiation, proliferation, and cytokine secretion [23, 28]. NFATc3 and NFATc2 promoted Th1 differentiation [29, 30]. Moreover, NFATc4 takes part in the thymic development and survival of T cells [30]. NFATc4 cooperated with NFATc1 and NFATc2 to induce different cytokines such as IL-2, IL-4, IFN-γ [30]. A significant reduction of NFATc3 expression occurred in CB T cells after treatment with K562-derived exosomes, whereas such variation did not confirm NFATc4 in the same condition. Thus, since NFATc3 expression induces the Th1 differentiation, the decrease of this transcription factor in T cells may suppress the cellular immune activation in the CML niche [29, 30].

Several studies showed the importance of different suppressor T cells, particularly regulatory T cells (T-regs) and IL-17 producing T-reg in the tumor microenvironment [18, 26]. The hallmarks of these cells include increased levels of FOXP3, secretion of anti-inflammatory cytokines, and sometimes the production of IL-6 and IL-17 [18, 26]. The results of our study highlighted the overexpression of FOXP3 as an essential transcription factor to the development of T-reg cells and their function. Also, a significant elevation of anti-inflammatory cytokines such as IL-10 besides a decrease of pro-inflammatory factor IL-1β may emphasize the ability of CML-derived exosomes in the development of immune suppressor T cells, especially T-regs. It is worth noting that K562-derived exosomes induced the IL-6 and IL-17 productions. So, these observations showed that CML-derived exosomes modified cytokine profiles of T cells toward FOXP3+ T cell-like cells [18]. Moreover, there were no significant changes in the expression of C-myc and PI3K involved in cell proliferation.

Some studies showed that reduction of ROS and increasing NO levels are related to the existence of tumor favorable IL-17 producing T cells [18]. The results derived from the current study showed a significant reduction in ROS levels in CB T cells exposed to K562-derived exosomes. Production of some inhibitory cytokines such as IL-10 and TGF-β beside IL-17 has been shown in p47phox deficient T cells, and ROS decrement induces Th17 cell differentiation [31]. Also, we observed a significant ascending in the transcription of NQO1, which has a protective mechanism against ROS production in cells, associated with the amount of intracellular ROS in treated T cells [32].

Jafarzadeh et al. revealed that different cells in the Bone-marrow niche exhibit diverse behaviors encounter with K562 derived exosomes that eventually lead to the development of cancer cells [9]. Furthermore, while BM-MSC increased NO production and reduced intracellular ROS levels, macrophages reduced the concentrations of both [9]. Production of NO is a way by which MSCs and tumoral cells can diminish the proliferation and immune functions of immune cells [9, 29]. Furthermore, increased NO levels in the serum of the patients with CML remarks its pathological and physiological importance [19]. The results derived from the current study showed that NO concentration was elevated in cord blood T cells after exposure to K562-derived exosomes. Enhancement of NO biosynthesis may occur due to nitric oxide synthase activity, which negatively influences the immune properties of T cells [19, 33]. Also, it has been reported that nitric oxide alters CD4 + CD25- effector cells to a population of CD4 + CD25 + Treg cells [19, 33].

Altogether, it seems reasonable to presume that K562 CML cell-derived exosomes accelerate the tumor favorable condition via modification of different stromal and immune cells in bone marrow niches such as BM-MSC, macrophages [9] as well as T cells.

## Conclusion

Regarding the results derived from the current study, K562 exosomes stimulated the immune suppressor functions in CB-derived T cells by inducting anti-inflammatory cytokines such as IL-10, reducting ROS levels, and arising of NO synthesis in these cells. Moreover, considering the elevated FOXP3, IL-6, and IL-17 levels in these cells, CML cell-derived exosomes may induce T cell fate toward tumor favorable T cells instead of conventional activated T cells (Th1, Th2, CTL, etc.) [18, 29]. Finally, it is worth mentioning that CML secretomes, particularly exosomes, require more comprehensive in vitro and in vivo niche-related investigations for planning impressive local niche-directed oncological therapies.

## Supplementary Information

**Additional file 1.**

## Data Availability

The data that support the findings of this study are available on request from the corresponding author. The data are not publicly available due to privacy or ethical restrictions.
