# Supplementary material for: CML derived exosomes promote tumor favorable functional performance in T cells
Source: BMC Cancer. 2021 Sep 7;21:1002. doi: 10.1186/s12885-021-08734-3 (PMC8424959; doi:10.1186/s12885-021-08734-3)

**Scanning electron microscopy (SEM)**

**Figure1. Shows the images of purified exosomes derived scanning electron microscopy (SEM) and Transmission electron microscopy (TEM).** **a;** Vesicles were isolated from the cell culture supernatant of K562 cells and examined by electron microscopy. **b;** Transmission electron microscopy (TEM) of K562-derived exosomes.

1a; Vesicles were isolated from the cell culture supernatant of K562 cells and examined by electron microscopy.


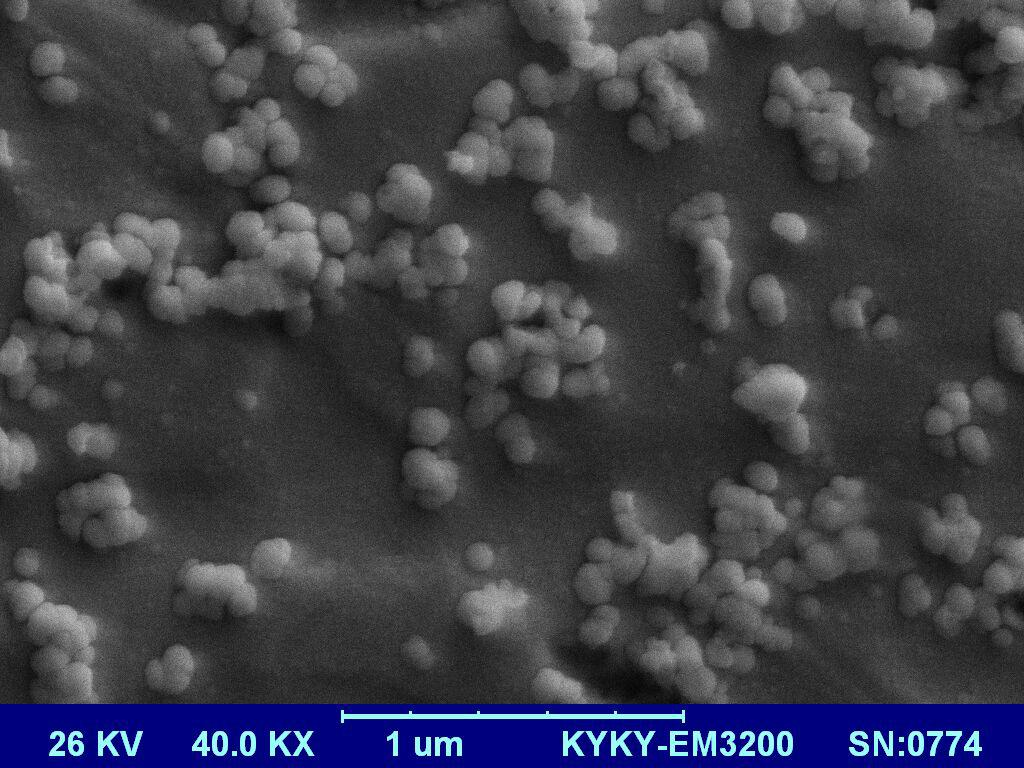


1b; Transmission electron microscopy (TEM) of K562-derived exosomes.
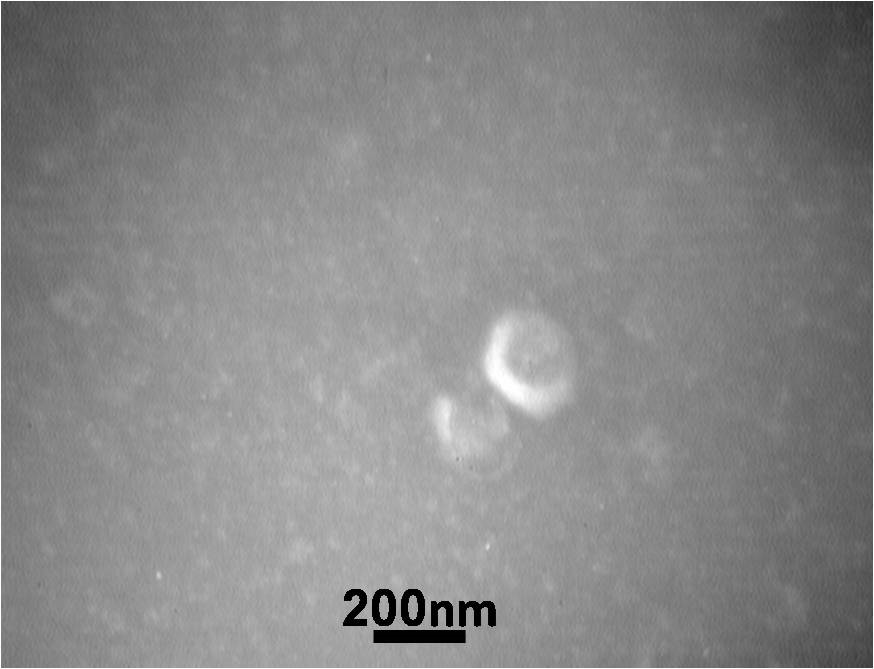


**Western blotting**

Western blotting was conducted to evaluate the expression of CD9, CD63, CD81 in K562 derived exosomes. Complete form of blotting gels was illustrated below (Figure1a-b).

**Figure2.** **Effects of miR-182 overexpression in NFATc3, FOXO1 and CD3d expression and FOXP3+ Tregs population differentiation**

**a;** Shows complete blotting of CD81, **b**; shows complete blotting of CD9, **c**; shows complete blotting of CD63.

**2a;** Shows complete blotting of CD81 (26KDa).


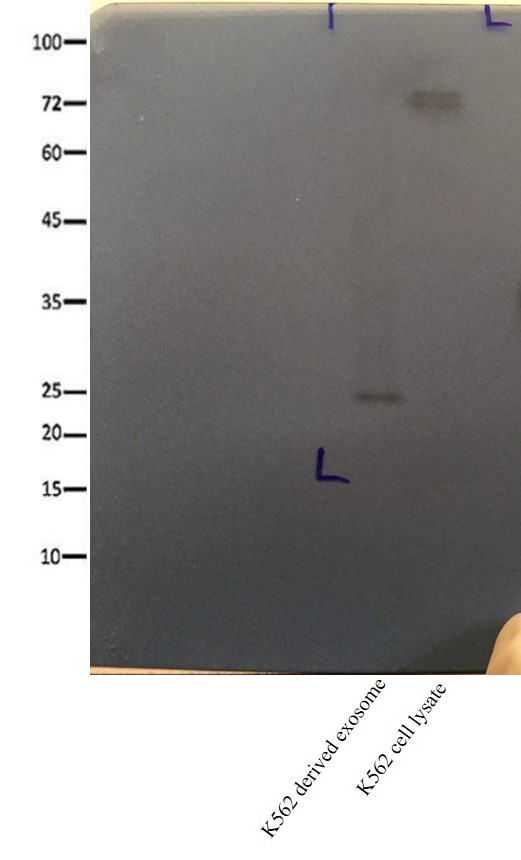


**2b;** Shows complete blotting of CD9 (24KDa).


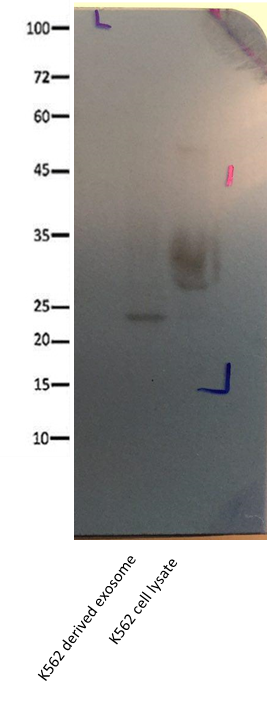


**2b;** Shows complete blotting of CD63.


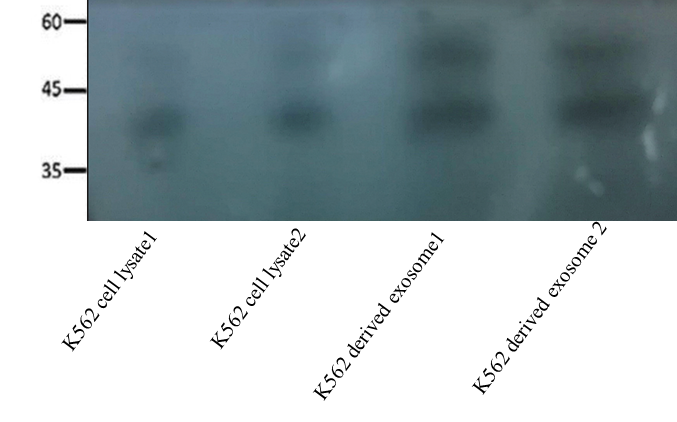

Supplement: Supplementary file 1 — Additional file 1. [file 12885_2021_8734_MOESM1_ESM.docx]
